# Real-world effectiveness of dupilumab in COPD: a German multicenter analysis

**DOI:** 10.1186/s12931-026-03940-8

**Published:** 2026-10-06

**Authors:** Mustafa Abdo, Dirk Skowasch, Timm Greulich, Nora Drick, Marek Lommatzsch, Ralf-Harto Hübner, Frederik Trinkmann, Katrin Milger, Jessica Rademacher, Konstantina Kontogianni, Maria Aetou, Michael Dreher, Peter Alter, Eva Pappe, Leonie Biener, Sebastian Sander, Jan Heyckendorf, Kathrin Kahnert, Jeremias Götschke, Claus Neurohr, Daiana Stolz, Vera Veith, Henrik Watz, Benjamin Waschki, Pontus Mertsch

**Affiliations:** 1 Department of Internal Medicine I, University Medical Center Schleswig-Holstein Campus Kiel, Airway Research Center North (ARCN), German Center for Lung Research (DZL), Arnold-Heller-Str 3, 24105 Kiel, Germany; 2 Department of Internal Medicine II, Cardiology, Pneumology, Angiology, University Hospital Bonn, Bonn, Germany; 3 Department of Respiratory, Intensive Care, and Sleep Medicine, Marburg University, Marburg, Germany; 4 German Center for Lung Research (DZL), Marburg, Germany; 5 Department of Respiratory Medicine and Infectious Diseases, Hannover Medical School, Biomedical Research in Endstage and Obstructive Lung Disease Hanover (BREATH), German Center for Lung Research (DZL), Hannover, Germany; 6 Department of Pneumology, University of Rostock, Rostock, Germany; 7 Department of Infectious Diseases, Respiratory Medicine and Critical Care, Charité - Universitätsmedizin Berlin, corporate member of Freie Universität Berlin and Humboldt- Universität zu Berlin, Berlin, Germany; 8 Department of Pneumology and Critical Care Medicine, Thoraxklinik Heidelberg, Heidelberg, Germany; 9 Division of Respiratory Medicine, Department of Internal Medicine, Lung Research Cluster, Medical University of Graz, Graz, Austria; 10 Department of Pneumology and Intensive Care Medicine, University Hospital Aachen, Aachen, Germany; 11 Department of Medicine V, LMU University Hospital, Comprehensive Pneumology Center (CPC-M), Member of the German Center for Lung Research (DZL), Munich, Germany; 12 Lungenärzte Vincentinum, Augsburg, Germany; 13 Clinic of Respiratory Medicine, University of Freiburg, Freiburg, Germany; 14 LungenClinic Grosshansdorf, Airway Research Center North (ARCN), German Center for Lung Research, Grosshansdorf, Germany; 15 Department of Respiratory Medicine, University Hospital Schleswig- Holstein Campus Luebeck, Airway Research Center North (ARCN), German Center for Lung Research, Campus Luebeck, Luebeck, Germany

**Keywords:** Dupilumab, Real-world, Exacerbation, COPD, Air trapping, Lung function

## Abstract

**Background:**

Dupilumab reduced exacerbations in patients with COPD and type 2 inflammation in clinical trials; however, multicenter real-world data remain limited.

**Methods:**

We performed a multicenter analysis of patients with COPD treated with dupilumab across centers in Germany. Patients with comorbid asthma were included to compare outcomes between COPD-only patients and those with comorbid asthma, as well as between patients with and without chronic bronchitis (CB). The primary endpoint was change in the annualized rate of moderate-to-severe exacerbations. Changes in lung function and CAT score were also assessed.

**Results:**

A total of 99 patients were included (60% male, mean age 66 years, FEV₁ 37% predicted, DLCO 41% predicted, 45% comorbid asthma, 58% CB, 7% current smokers). Over a median follow-up of 11 months [IQR 8–14], dupilumab reduced the annualized moderate-to-severe exacerbation rate from 3.52 (95% CI 2.94–4.09) at baseline to 1.04 (95% CI 0.71–1.37) during follow-up, corresponding to a 70% reduction. Reductions in exacerbation rates were consistent irrespective of exacerbation severity, comorbid asthma, CB, or emphysema severity. CAT improved by a mean of 4.5 points. FEV₁ improved by 50 mL (*p* = 0.077), while greater improvements were observed for residual volume (− 150 mL; *p* = 0.071) and total airway resistance (− 12.6%; *p* < 0.001). Eight patients were non-responders and had no reduction in exacerbation under treatment.

**Conclusion:**

In this real-world multicenter cohort, dupilumab effectively reduced exacerbation burden in patients with COPD and type 2 inflammation. Exploratory subgroup analyses suggested consistent treatment effects across different clinical phenotypes, although these findings require confirmation in larger studies. The high baseline exacerbation burden may partly explain the marked treatment response.

## Introduction

Chronic obstructive pulmonary disease (COPD) is a prevalent yet underrecognized global health burden [1] and one of the leading causes of morbidity and mortality worldwide [2]. Beyond chronic respiratory symptoms and progressive lung function loss, acute exacerbations represent key clinical events, as they are associated with increased hospitalization rates, impaired health-related quality of life, and increased short- and long-term mortality [3, 4]. Accordingly, prevention of moderate and severe exacerbations constitutes a major therapeutic goal in COPD management [3].

COPD exacerbations are biologically heterogeneous and may be triggered by respiratory infections, inhaled environmental exposures, or dysregulated host inflammatory response [5, 6]. Among these, type 2 (T2) inflammation contributes to exacerbation susceptibility in a clinically relevant subgroup of patients with COPD. Blood eosinophil count and fractional exhaled nitric oxide (FeNO) are accessible biomarkers of T2 inflammation, that allow a targeted therapy approach in COPD [6–8].

Dupilumab, a monoclonal antibody targeting IL-4/IL-13 signaling, has recently been approved for the treatment of patients with COPD with recurrent exacerbations and increased marker of T2 inflammation. Subsequently, its efficacy was demonstrated in two large, randomized placebo-controlled trials in this population [9, 10]. In both studies, dupilumab consistently reduced moderate and severe exacerbations by 30–34%, representing a clinically meaningful treatment effect [9, 10]. Moreover, pooled analyses from both studies demonstrated reductions in emergency department visits and hospital admissions, while subjects experiencing exacerbation required fewer courses of systemic corticosteroids under dupilumab treatment [11]. Dupilumab was also associated with improvements in lung function, indicated by pre-bronchodilator FEV₁, and patient-reported outcomes [9, 10].

Although these findings provide evidence supporting dupilumab therapy in patients with COPD and T2 inflammation, data on the real-world effectiveness in this patient population remain scarce. Therefore, the present multicenter analysis aimed to (1) evaluate the effectiveness of dupilumab treatment in COPD subjects in routine clinical care, (2) investigate treatment response across clinically relevant respiratory phenotypes including comorbid asthma, chronic bronchitis, and emphysema, and (3) assess changes in lung function and physiological markers of small airway dysfunction, including air trapping and airway resistance.

## Methods

### Study design and population

This retrospective multicenter study evaluated patients with COPD receiving dupilumab for recurrent exacerbations and evidence of type 2 inflammation in routine clinical practice across specialized respiratory centers in Germany. COPD diagnosis and indication for dupilumab therapy were established locally by experienced treating physicians according to clinical assessment and national prescribing practice. Anonymized clinical data were collected from 14 participating centers. Patients within or beyond their sixth month of follow-up after treatment initiation were included to assess treatment effectiveness on exacerbation reduction. The study was approved by the ethics committee at the University of Lübeck School of Medicine (Lübeck, Germany; Az. 2026-085).

### Primary endpoint

The primary outcome was the change in annualized moderate-to-severe exacerbation rate under dupilumab treatment. For all patients, baseline exacerbation burden was assessed based on the number of exacerbations during the whole 12 months preceding treatment initiation. To account for variable follow-up duration under treatment, exacerbation rates during follow-up were normalized to a 12-month period. Moderate exacerbations were defined as events requiring burst systemic corticosteroid treatment and/or antibiotic therapy, whereas severe exacerbations were defined as exacerbations requiring hospitalization. Although the current GOLD recommendations incorporate the Rome proposal for exacerbation severity assessment [3], the treatment-based classification was used in the present study because it aligns with the definitions used in the pivotal phase 3 dupilumab trials (BOREAS and NOTUS), and the data required to apply the Rome proposal were not systematically collected in this retrospective real-world cohort. Separate analyses were additionally performed for moderate and severe exacerbations individually. As an exploratory analysis, subjects with an unchanged or increased annualized moderate-to-severe exacerbation rate during dupilumab treatment compared with baseline were classified as non-responders.

### Clinical data and subgroup analyses

Further clinical data, including demographics, inhaled and oral corticosteroid therapy, exacerbation history, symptoms, and physician-reported comorbid respiratory diseases such as emphysema, bronchiectasis, and asthma, were collected from the participating centers. Clinical characteristics and phenotypes were assigned locally by experienced treating respiratory physicians according to routine clinical assessment and recommendations of international guidelines. Subgroup analyses were subsequently performed to compare reductions in annualized exacerbation rates across these clinical phenotypes.

### Lung function, type 2 biomarkers, and symptom assessment

Pre-bronchodilator spirometry and body plethysmography were performed according to ERS/ATS recommendations at the participating centers [12, 13]. Lung function data collected at treatment initiation and at follow-up included FEV₁, FEV₁/FVC ratio, residual volume, and total specific airway resistance (sRtot). In addition, type 2 inflammatory biomarkers including blood eosinophil counts and fractional exhaled nitric oxide (FeNO), when available, were collected. Changes in lung function parameters and type 2 biomarkers during dupilumab treatment were assessed as secondary endpoints. COPD Assessment Test (CAT) scores were also evaluated, including the proportion of subjects achieving an improvement exceeding the minimal clinically important difference (MCID) [14].

### Statistical analysis

Exacerbation rates were annualized by accounting for individual follow-up duration under dupilumab treatment. Baseline exacerbation frequency reflected the preceding 12 months, whereas follow-up exacerbation rates were calculated as the number of exacerbations during follow-up divided by follow-up duration in months and annualized to 12 months. Analyses were performed separately for moderate-to-severe, moderate, and severe exacerbations. Mean annualized exacerbation rates before and during dupilumab treatment, as well as percentage reduction, were calculated for each category.

Subgroup analyses were additionally performed according to the presence of chronic bronchitis symptoms, comorbid asthma, frequent exacerbations, nocturnal awakening symptoms, systemic corticosteroid use, and severely reduced diffusing capacity (DLCO < 40% predicted). For each subgroup, annualized exacerbation rates before and during treatment and percentage reduction were calculated. Differences in exacerbation reduction between subgroups were assessed using multivariable linear regression models adjusted for age, sex, body mass index, blood eosinophil count, and baseline FEV₁ % predicted.

Changes in CAT score and lung function parameters were assessed using paired analyses. Depending on the distribution of the change scores, paired t-tests or Wilcoxon signed-rank tests were applied. Mean changes and percentage changes from baseline were calculated for all parameters.

All statistical analyses were performed using RStudio (version 2025.05.1). A two-sided p-value < 0.05 was considered statistically significant.

## Results

A total of 99 patients with COPD receiving dupilumab treatment and with at least 6 months of follow-up were included in the analysis (Table 1). Patients had a mean age of 66 ± 8 years, were predominantly male, and the majority were former smokers. Nearly all patients received background inhaled triple therapy. Most of the patients had emphysema, while almost half had comorbid asthma and more than half reported symptoms consistent with chronic bronchitis (Table 1). Overall, the patients had marked lung function impairment, indicated by marked airflow obstruction and severely reduced diffusing capacity (Table 1). Both baseline blood eosinophil count and FeNO were elevated (Table 1). The patients also had a high exacerbation burden, with a mean annualized moderate-to-severe exacerbation rate of 3.2 ± 2.7 events in the preceding 12 months. Symptom burden was likewise high, reflected by elevated CAT scores (Table 1).

**Table 1:** Baseline patients‘ characterstics

Characteristic	Value
Age, years	66 ± 8.3
Male sex, %	60%
BMI, kg/m²	26.6 ± 5.4
Current smokers, %	7%
Triple inhaled therapy, %	99%
Current treatment with systemic corticosteroid, %	24%
Emphysema, %	83%
Comorbid asthma, %	45%
Night-time awakening symptoms, %	29%
Chronic bronchitis, %	58%
Allergic rhinitis, %	9%
Bronchiectasis, %	11%
FEV₁/FVC	0.48 ± 0.12
FEV₁ % predicted	37 ± 13
DLCO % predicted	41 ± 16
Blood eosinophils, cells/µL ≥ 300/µl, % < 300/µl, %	355 ± 286, 300 (190–460) 55% 45%
FeNO, ppb ≥ 25 ppb, % < 25ppb, %	35 ± 31, 25 (13–55) 53% 47%
CAT score	25 ± 8
Moderate exacerbation past 12 months	2.7 ± 3.1 2 (1–3)
Severe exacerbation past 12 months	0.83 ± 1.2 0.0 (0.0–1.0)
Moderate or severe exacerbation past 12 months	3.2 ± 2.7 3 (2–4)

Values are presented as mean ± SD, or median (interquartile range) or n (%). BMI = body mass index; FEV₁ = forced expiratory volume in 1 s; FVC = forced vital capacity; CAT = COPD Assessment Test; FeNO = fractional exhaled nitric oxide; DLCO = diffusing capacity of the lung for carbon monoxide. Blood eosinophil counts and FeNO values represent the last available measurements before treatment initiation. Blood eosinophil data were available for 89 subjects and FeNO data for 51 subjects

**Fig. 1:**
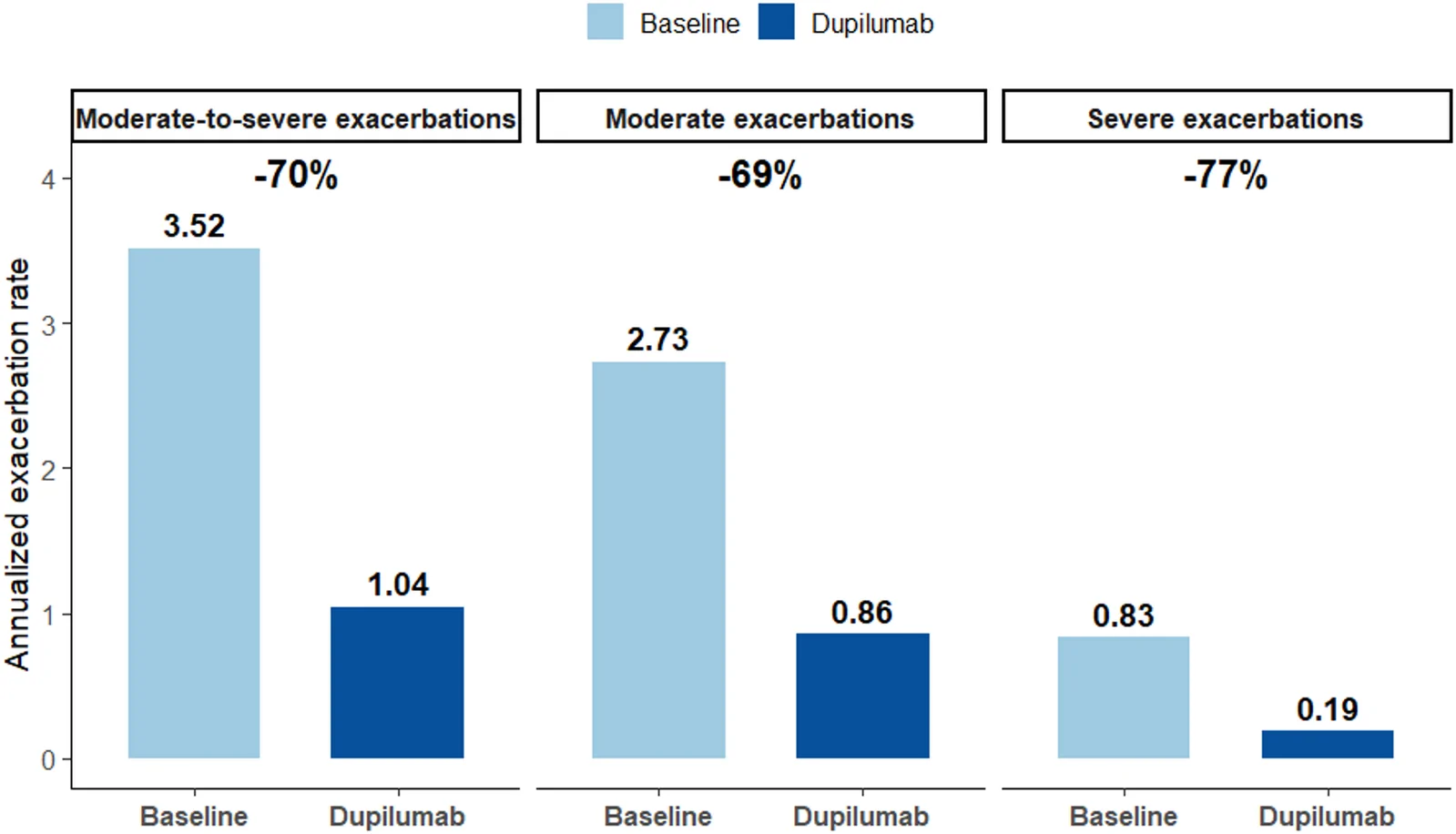
Annualized exacerbation rates at baseline and during dupilumab treatment according to exacerbation severity, with percentage reduction in exacerbation rates during follow-up

During a median follow-up of 11 months (IQR 8–14), dupilumab treatment was associated with marked reductions in exacerbation burden across all exacerbation severity categories (Fig. 1). The annualized rate of moderate-to-severe exacerbations decreased from 3.52 events/year (95% CI 2.94–4.09) at baseline to 1.04 events/year (95% CI 0.71–1.37) during follow-up, corresponding to a 70% reduction. Similarly, annualized moderate exacerbation rates decreased from 2.73 (95% CI 2.12–3.34) to 0.86 events/year (95% CI 0.53–1.19), reflecting a 69% reduction, while severe exacerbation rates declined from 0.83 (95% CI 0.60–1.07) to 0.19 events/year (95% CI 0.08–0.30), corresponding to a 77% reduction (Fig. 1).

**Fig. 2:**
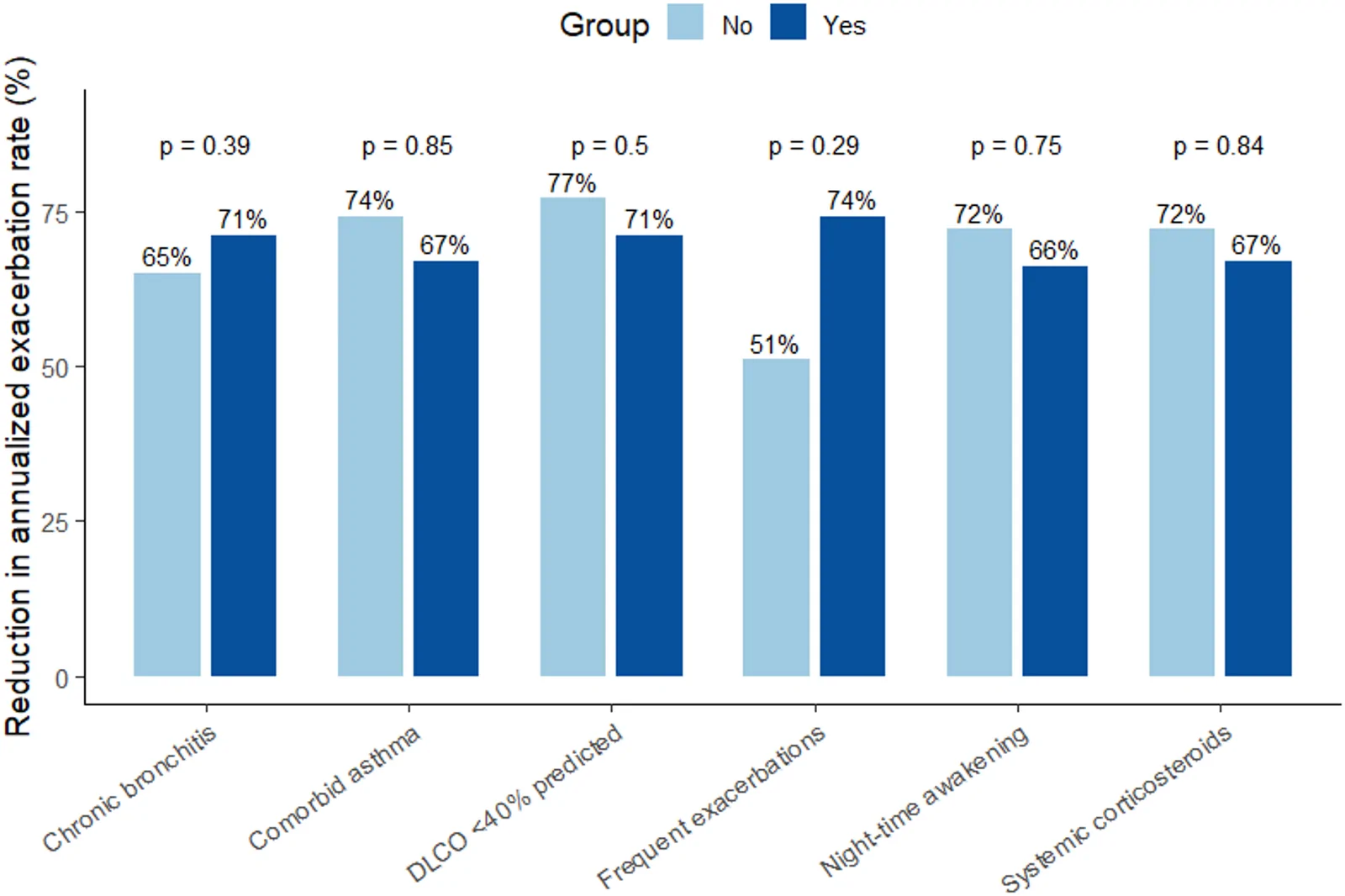
Reduction in annualized moderate-to-severe exacerbation rates according to clinically relevant COPD phenotypes and baseline characteristics

Subgroup analyses demonstrated consistent reductions in annualized exacerbation rates across clinically relevant COPD phenotypes after adjustment for age, sex, body mass index, baseline blood eosinophil count and FEV₁ % predicted, and follow-up duration (Fig. 2). Comparable treatment responses were observed irrespective of chronic bronchitis symptoms, comorbid asthma, nocturnal awakening symptoms, or current treatment with systemic corticosteroid, with no significant differences identified between subgroups after multivariable adjustment (Fig. 2). Given the high prevalence of emphysema within the cohort, an additional analysis using severely reduced diffusing capacity as a quantitative surrogate marker of emphysema severity demonstrated similarly consistent treatment effects. Subjects with DLCO < 40% predicted (*n* = 36/73) exhibited a 71% reduction in exacerbation burden compared with 77% in subjects with higher DLCO values (adjusted *p* = 0.50). Similarly, subjects with frequent exacerbations, defined as ≥ 3 exacerbations during the preceding 12 months (*n* = 58/99), demonstrated numerically greater reduction in exacerbation burden compared with less frequent exacerbators (74% vs. 51%), although this difference did not reach statistical significance after multivariable adjustment (adjusted *p* = 0.29).

In an exploratory analysis stratified by baseline blood eosinophil count, the annualized exacerbation rate decreased from 3.21 to 1.36 events/year (58% reduction) in patients with < 300 cells/µL and from 4.11 to 0.91 events/year (78% reduction) in those with ≥ 300 cells/µL. After adjustment for age, sex, body mass index, and FEV₁ % predicted, the difference between groups was not statistically significant (adjusted *p* = 0.086).

Markers of type 2 inflammation also improved during dupilumab treatment. Among subjects with available paired measurements at baseline and follow-up, median FeNO levels decreased from 27.0 (IQR, 18.0–55.8) ppb at baseline to 15.0 (IQR, 9.5–24.5) ppb during follow-up, corresponding to a median reduction of 14.5 ppb (IQR, − 34.0 to − 2.8; *p* < 0.001, *n* = 36). Median blood eosinophil counts decreased from 300.0 (IQR, 190.0–475.0) to 230.0 (IQR, 125.0–400.0) cells/µL, reflecting a median reduction of 60.0 cells/µL (IQR, − 270.0 to 100.0; *p* = 0.051, *n* = 63).

Dupilumab treatment was associated with modest, yet consistent improvements across lung function parameters. FEV₁ increased from 1.04 ± 0.43 L at baseline to 1.09 ± 0.49 L during follow-up, corresponding to a mean improvement of 50 ml (+ 4.8%; *p* = 0.077). Residual volume decreased from 4.49 ± 1.24 L to 4.34 ± 1.16 L, reflecting a mean reduction of 150 ml (− 3.2%; *p* = 0.071). Total specific airway resistance (sRtot, kPa·s) showed significant improvement, decreasing from 3.85 ± 2.78 to 3.36 ± 2.36 kPa·s, corresponding to a mean reduction of 0.49 kPa·s (− 12.6%; *p* < 0.001).

**Fig. 3:**
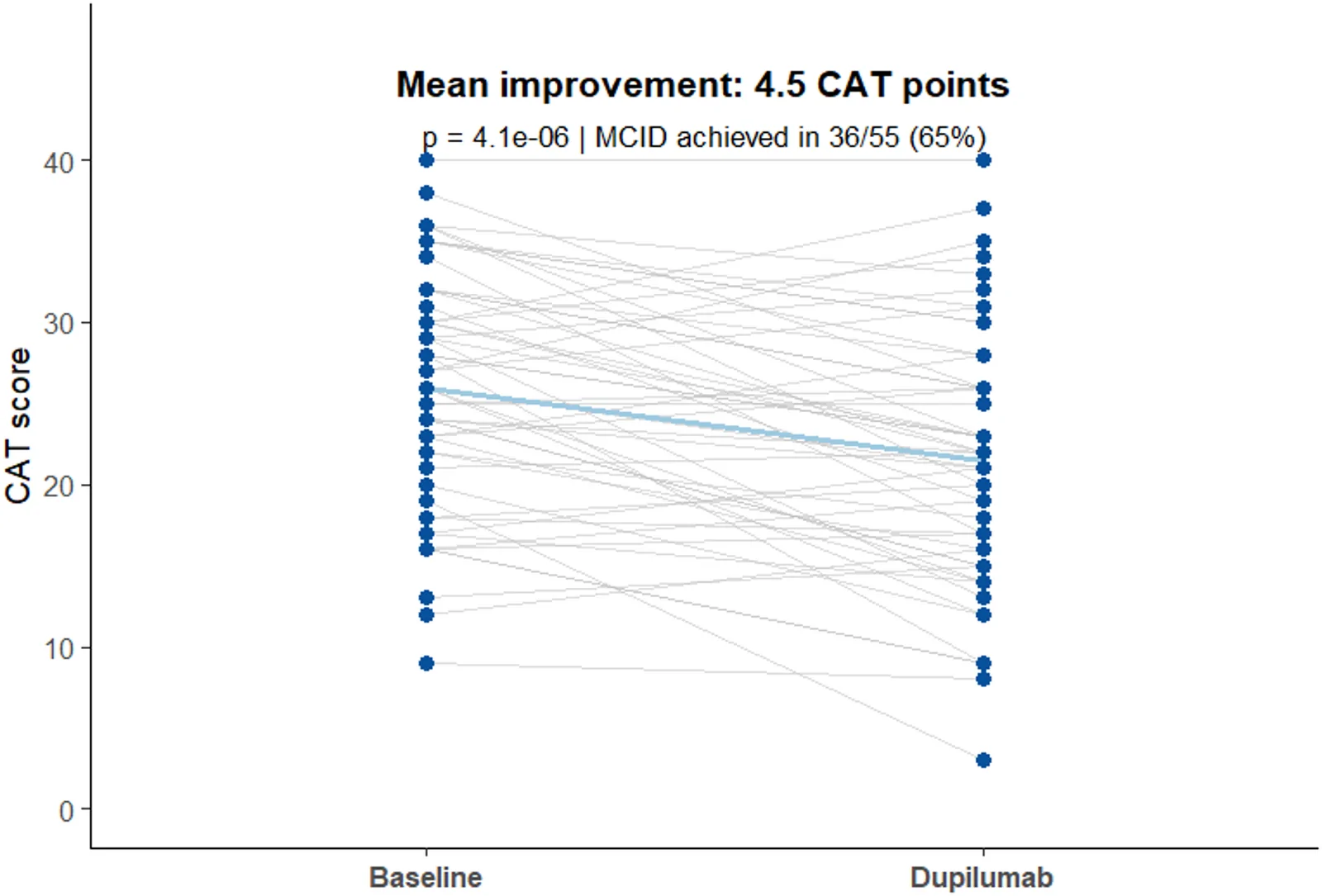
Changes in COPD Assessment Test (CAT) score from baseline to follow-up during dupilumab treatment. Dark blue dots represent individual baseline and follow-up CAT scores, and the light blue line indicates the mean change during treatment

CAT scores improved significantly during dupilumab treatment (Fig. 3). Mean CAT score decreased by 4.5 points from baseline to follow-up (*p* < 0.001), exceeding the established minimal clinically important difference (MCID). Overall, 65% of subjects achieved a clinically meaningful improvement of at least 2 CAT points under treatment.

Despite the overall marked reduction in exacerbation burden, 8 subjects (8%) had an unchanged or increased annualized moderate-to-severe exacerbation rate under dupilumab. All non-responders were male former smokers. These subjects had a mean age of 61 ± 7 years, baseline FEV₁ of 41.5 ± 15.0% predicted, and DLCO of 53.0 ± 24.9% predicted. Baseline T2 markers were elevated, with blood eosinophils of 275 ± 196 cells/µL and FeNO of 34.8 ± 27.9 ppb. Most non-responders had comorbid asthma (7/8), while chronic bronchitis symptoms were present in 4/8 subjects and current treatment with systemic corticosteroid in 3/8. During a median follow-up of 17 months, annualized moderate-to-severe exacerbation rates increased from 2.5 events/year (95% CI 1.5–3.5) at baseline to 3.3 events/year (95% CI 1.9–4.6) during follow-up.

Possible treatment-related adverse events were reported in 7 subjects and ranged from conjunctivitis/dry eye and skin-related symptoms to musculoskeletal pain, headache, and abdominal discomfort. None led to treatment discontinuation.

## Discussion

In this real-world cohort of patients with COPD and type 2 inflammation receiving triple inhaled therapy, treatment with dupilumab was associated with a marked reduction in annualized exacerbation rates over a median follow-up time of 11 months. Annualized moderate-to-severe exacerbation rates decreased by approximately 70%, with consistent reductions observed across moderate and severe exacerbations. Importantly, treatment responses were broadly consistent across clinically relevant COPD phenotypes, including subjects with emphysema, chronic bronchitis, comorbid asthma, and frequent exacerbations. Dupilumab treatment was additionally associated with improvements in respiratory symptoms, airway resistance, and biomarkers of type 2 inflammation, whereas changes in spirometry were modest. Despite the overall favorable treatment response, a small subgroup of subjects did not demonstrate exacerbation reduction under treatment, underscoring a possible heterogeneous clinical response to dupilumab in COPD with type 2 inflammation.

The magnitude of exacerbation reduction observed in our real-world cohort appeared greater than that reported in the phase 3 BOREAS and NOTUS trials, which demonstrated approximately 30–34% reductions in annualized moderate-to-severe exacerbation rates under dupilumab treatment compared to placebo [9, 10]. It should be noted that these trials assessed placebo-adjusted treatment effects, whereas our analysis compared exacerbation rates before and after dupilumab initiation. Importantly, subjects in our cohort had a higher baseline exacerbation burden, with a mean annualized moderate-to-severe exacerbation rate of 3.2 ± 2.7 events during the preceding 12 months and an observed baseline annualized exacerbation rate of 3.5 events/year, compared with a mean number of exacerbations during the preceding 12 months of 2.1 ± 0.9 and 2.3 ± 1.0 in NOTUS and BOREAS, respectively [9, 10]. Prior exacerbation history is the strongest predictor of future exacerbation risk in COPD [15], and the high baseline exacerbation burden observed in our cohort may therefore partly contribute to the pronounced absolute reduction in exacerbation frequency observed during dupilumab treatment. In line with this observation, subjects with frequent exacerbations at baseline (≥ 3 exacerbations during the preceding 12 months) demonstrated numerically greater reductions in exacerbation burden compared with less frequent exacerbators (74% vs. 51%), although this difference did not reach statistical significance after multivariable adjustment. Our findings are also directionally consistent with a recently published smaller real-world cohort of 23 subjects with COPD treated with dupilumab, in which annualized exacerbation rates decreased from 3.47 to 1.55 events/year, corresponding to a 55% reduction [16]. Together, these findings suggest that our cohort represented a population with particularly high exacerbation burden and advanced disease severity despite optimized inhaled therapy.

Notably, exacerbation reductions in our study were consistently observed across both moderate and severe exacerbations, with severe exacerbations decreasing by 77% during follow-up. This observation is of particular interest given that the pooled analysis of BOREAS and NOTUS did not demonstrate a statistically significant reduction in annualized severe exacerbation rates under dupilumab treatment (0.084 vs. 0.124 events/year; rate ratio 0.674, 95% CI 0.438–1.037; *p* = 0.073) [17]. The comparatively lower baseline burden of severe exacerbations in the phase 3 populations, for example the 0.3 ± 0.7 severe exacerbations during the preceding year reported in BOREAS, may partly explain these differences.

Whether differences in smoking status may also have contributed to the marked reduction in annualized moderate-to-severe exacerbation rates observed in our cohort remains unclear. In contrast to BOREAS and NOTUS, in which approximately 30% of subjects were current smokers [17], the vast majority of subjects in our cohort were former smokers. However, pooled analyses of the phase 3 trials demonstrated comparable dupilumab efficacy in current smokers (rate ratio 0.683, 95% CI 0.528–0.884) and former smokers (rate ratio 0.685, 95% CI 0.576–0.815) [17]. Nevertheless, active cigarette smoke exposure exerts complex immunomodulatory effects on the airway, including altered epithelial and innate immune responses as well as enhanced neutrophilic inflammation [18]. Smoking has also been associated with altered responsiveness to anti-inflammatory therapies in COPD [18] and may influence upstream inflammatory signaling pathways involved in disease pathobiology [19]. Although the impact of active smoking on IL-4/IL-13-targeted therapy remains incompletely understood, the potential interaction between smoking-related immune dysregulation and biologic treatment response warrants further investigation.

A further clinically relevant difference from the pivotal phase 3 trials is the inclusion of subjects with comorbid asthma in our real-world cohort. Both BOREAS and NOTUS excluded patients with a current diagnosis or history of asthma [9, 10], whereas almost half of the subjects in our cohort had comorbid asthma. To further capture clinical features suggestive of asthma beyond physician diagnosis alone, we additionally evaluated nocturnal awakening symptoms, which likewise did not identify differential treatment responses. These findings suggest that the observed exacerbation reduction was not restricted to a specific asthma-overlap phenotype. This observation is noteworthy given that asthma is typically characterized by pronounced type 2 inflammation, which could theoretically favor enhanced responsiveness to IL-4/IL-13 blockade. In this context, it is notable that 7 of the 8 non-responders in our cohort had comorbid asthma. Although these findings should be interpreted cautiously given the small sample size, they highlight the need for further studies investigating biologic treatment responses in patients with coexisting COPD and asthma.

Furthermore, BOREAS and NOTUS largely focused on patients with COPD and chronic bronchitis features [9, 10], a phenotype characterized by high symptom burden and increased exacerbation risk [20]. Chronic bronchitis might also be mechanistically relevant for IL-4/IL-13-targeted therapy because the IL-13 axis is closely linked to goblet-cell hyperplasia and mucus hypersecretion [21]. In our cohort, however, nearly half of the subjects did not report chronic bronchitis symptoms, and reductions in exacerbation burden were comparable irrespective of chronic bronchitis status. These findings suggest that dupilumab responsiveness may extend beyond the chronic bronchitis phenotype in COPD.

Another notable characteristic of our cohort was the high prevalence of emphysema. Because emphysema was primarily physically reported, we additionally used severely reduced diffusing capacity (DLCO < 40% predicted) as a surrogate marker of advanced emphysema severity. Dupilumab-associated reductions in exacerbation burden were comparable between subjects with severely impaired and preserved diffusing capacity, without significant subgroup differences. These findings are consistent with pooled analyses from BOREAS and NOTUS, in which dupilumab reduced exacerbation rates by 29% and 31% in subjects with and without emphysema, respectively, without evidence of treatment interaction according to emphysema status [22].

Although 45% of patients had a baseline blood eosinophil count < 300 cells/µL, the reported value represented the measurement before treatment initiation and did not necessarily correspond to the value on which the treatment indication was based. Notably, the baseline blood eosinophil distribution in our cohort was comparable to that reported at randomization in the BOREAS and NOTUS trials [9, 10]. In an exploratory subgroup analysis, patients with baseline blood eosinophil counts ≥ 300 cells/µL demonstrated a numerically greater reduction in exacerbation rate than those with lower eosinophil counts, consistent with previous analyses of the BOREAS and NOTUS trials showing that higher baseline type 2 inflammatory biomarkers are associated with greater reduction of exacerbation rate under dupilumab [23].

In contrast to the marked reduction in exacerbation burden, improvements in spirometric indices were more modest in our cohort, with FEV₁ increasing by approximately 50 ml during follow-up. This was lower than the improvements reported in BOREAS and NOTUS, in which prebronchodilator FEV₁ increased by 160 ml and 139 ml at week 12, respectively [9, 10]. One possible explanation is the greater degree of baseline airflow obstruction in our cohort, with a mean FEV₁ of approximately 37% predicted compared with nearly 50% predicted in the phase 3 populations. Despite the limited spirometric improvement, we observed numerically greater reductions in air trapping, reflected by a 150 ml decrease in residual volume, together with a significant reduction in total specific airway resistance. These findings suggest treatment effects at the level of the small airways, which represent the primary site of inflammation and airflow limitation in COPD [24]. In this context, type 2 inflammation has previously been linked to small airway dysfunction and air trapping independently of spirometric impairment [25, 26]. Further investigation into the effects of dupilumab on small airway dysfunction in COPD therefore is of interest.

Beyond exacerbation reduction, dupilumab treatment was also associated with clinically meaningful improvements in patient-reported outcomes, with mean CAT scores improving beyond the established minimal clinically important difference [14]. Although BOREAS and NOTUS assessed health status using the St. George’s Respiratory Questionnaire rather than CAT, both phase 3 trials likewise demonstrated significant improvements in respiratory health-related quality of life under dupilumab treatment [9, 10]. These findings support a consistent beneficial effect of dupilumab on patient-reported outcomes in COPD with type 2 inflammation.

Several limitations should be acknowledged. First, this was a retrospective real-world analysis without a control group, raising the possibility of regression to the mean due to the preferential selection of subjects with high exacerbation burden for biologic treatment. Second, several subgroup analyses were exploratory and based on relatively small patient numbers and should therefore be interpreted cautiously. Third, the diagnosis of respiratory comorbidities and clinical phenotypes, including emphysema and chronic bronchitis, was based on physician assessment and symptom reporting rather than standardized imaging or biomarker-based definitions. Finally, detailed information on inhaled corticosteroid therapy duration, adherence to inhaled therapy, and changes in oral corticosteroid exposure, which may influence exacerbation risk, was not systematically documented and therefore could not be evaluated.

In conclusion, this real-world study demonstrates that dupilumab was associated with marked reductions in exacerbation rates and clinically meaningful improvement in symptom burden in patients with COPD and type 2 inflammation despite advanced disease severity and high baseline exacerbation burden. These findings extend phase 3 trial evidence to a broader and more heterogeneous real-world COPD population and support further investigation of dupilumab across clinically relevant COPD phenotypes.

## Data Availability

The data analyzed in this study are not publicly available. The analyses were conducted using anonymized data, and patient consent for sharing individual-level data beyond the purposes of this study was not obtained.

## Declarations

### Generative AI and AI-assisted technologies in the manuscript preparation process

During the preparation of this work the author(s) used ChatGPT in order to check for writing, grammar, and formulation of language. After using this tool/service, the author(s) reviewed and edited the content as needed and take(s) full responsibility for the content of the published article.

## Abbreviations

CAT: COPD Assessment Test
COPD: Chronic Obstructive Pulmonary Disease
DLCO: Diffusing Capacity of the Lung for Carbon Monoxide
ERS/ATS: European Respiratory Society/American Thoracic Society
FeNO: Fractional Exhaled Nitric Oxide
FEV₁: Forced Expiratory Volume in 1 s
FEV₁/FVC: Ratio of Forced Expiratory Volume in 1 s to Forced Vital Capacity
MCID: Minimal Clinically Important Difference
sRtot: specific Total Airway Resistance
T2: Type 2 Inflammation
